# International Spread of MDR TB from Tugela Ferry, South Africa

**DOI:** 10.3201/eid1711.110291

**Published:** 2011-11

**Authors:** Graham S. Cooke, R. Kate Beaton, Richard J. Lessells, Laurence John, Simon Ashworth, Onn Min Kon, O. Martin Williams, P. Supply, P. Moodley, Alexander S. Pym

**Affiliations:** Imperial College, London, UK (G.S. Cooke); University of KwaZulu-Natal, Mtubatuba, South Africa (G.S. Cooke, R.J. Lessells); National Mycobacterium Reference Laboratory, London (R.K. Beaton); Imperial College National Health Service Healthcare Trust, London (L. John, S. Ashworth, O.M. Kon); Health Protection Agency Regional Laboratory Services, Bristol, UK (O.M. Williams); University of Lille–Nord de France, Lille, France (P. Supply); University of KwaZulu-Natal, Durban, South Africa (P. Moodley); Medical Research Council, Durban (A.S. Pym)

**Keywords:** MDR TB, HIV, health care workers, rpoB, antimicrobial resistance, South Africa, tuberculosis and other mycobacteria, multidrug resistance

## Abstract

We describe a death associated with multidrug-resistant tuberculosis and HIV infection outside Africa that can be linked to Tugela Ferry (KwaZulu-Natal, South Africa), the town most closely associated with the regional epidemic of drug-resistant tuberculosis. This case underscores the international relevance of this regional epidemic, particularly among health care workers.

Multidrug-resistant (MDR) and extensively drug-resistant (XDR) tuberculosis (TB) pose an increasing challenge to international health (*1*), particularly in the context of HIV infection. An outbreak of XDR TB around a rural hospital in Tugela Ferry (KwaZulu-Natal Province, South Africa) in 2006 received widespread international attention, in part because of the high case-fatality rate. More recent work has highlighted the risk for MDR TB among health care workers (*2*) in South Africa. We report a death outside of Africa associated with MDR TB that can be directly related to the epidemic of drug resistance in Tugela Ferry, the center most closely associated with the epidemic.

## The Study

A 42-year-old South Africa–born health care worker was admitted to a regional hospital in the United Kingdom with a 1-month history of fever, cough, and weight loss associated with cervical lymphadenopathy, choroidal tuberculoma (Figure), and pleural effusion. The patient had no history of TB treatment and no known family contact with TB. He had moved to the United Kingdom 6 years earlier. Extrapulmonary TB diagnosis was based on microscopic examination of a cervical lymph node specimen, and co-infection with HIV was identified (CD4 count 5 cells/µL).

**Figure:**
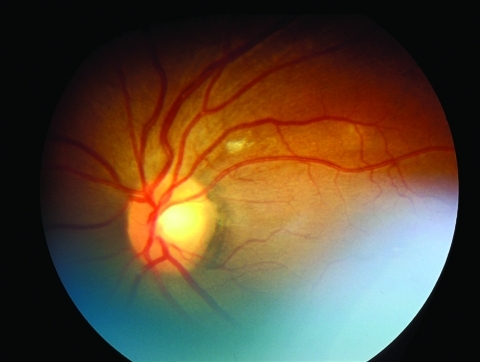
Retinal image from patient with evidence of choroidal tuberculosis.

After arrival in the United Kingdom, the patient worked as a temporary nurse in several health care facilities. Before that, the patient worked at the Church of Scotland Hospital in Tugela Ferry during 1996–2002 in general medical wards. This facility was the focus of the 2006 report of XDR TB, and most MDR isolates identified there during 2005–2007 were XDR TB (*3*).

A presumptive diagnosis of MDR TB was made 7 days after the patient sought care at the hospital. A line probe assay (INNO-LiPA Rif.TB; Innogenetics, Ghent, Belgium) performed on cervical lymph node aspirates identified *Mycobacterium tuberculosis* and a hybridization pattern consistent with *rpoB* gene mutation (associated with rifampin resistance and a high risk for MDR TB). Treatment was altered from weight-appropriate doses of rifampin, isoniazid, pyrazinamide, and ethambutol to include levofloxacin, amikacin, cycloserine, and protionamide. Because of known sensitivity patterns from isolates at Tugela Ferry and the possibility of XDR TB, para-aminosalicylic acid and linezolid were added to treatment, and intravenous amikacin was changed to capreomycin. Treatment was subsequently changed on the basis of culture results. The patient required 35 days of mechanical ventilation for likely pulmonary immune reconstitution syndrome after initiation of antiretroviral treatment. Although discharged from intensive care after successful treatment of TB, the patient remained hospitalized and died 90 days after first seeking care. Samples cultured from >1 site showed evidence of widespread MDR TB. More than 500 potentially infectious contacts were identified, but no secondary cases of TB have been diagnosed.

Culture confirmed the *M. tuberculosis* isolate from the patient to be resistant to rifampin, isoniazid, pyrazinamide, and ethambutol but sensitive to amikacin, capreomycin, moxifloxacin, para-aminosalicylic acid, and linezolid. These resistance and sensitivity characteristics identified the isolate as MDR TB rather than an XDR TB strain. Automated sequencing of *rpoB* revealed the L533P mutation previously associated with rifampin resistance (*4**–**6*) but not the D516G mutation found in XDR TB strains previously isolated from patients at Church of Scotland Hospital and from other hospitals within KwaZulu-Natal (*7*).

To explore whether the patient’s isolate was related to the F15/LAM4/KZN strain genotypes associated with the Tugela Ferry outbreak (*8*) and the broader population of drug-resistant strains in the region, we performed mycobacterial interspersed repetitive units (MIRU)–variable number tandem repeats (VNTR) typing and spoligotyping (*9*). The isolate displayed a typical LAM4 spoligotype (1111111111111111111100001111111100001110111), consistent with a F15/LAM4/KZN genotype. In the absence of full MIRU-VNTR typing for Tugela Ferry strains, the alleles of 15 loci were compared with those inferred by in silico analysis of publicly available genome sequences for five F15/LAM4/KZN strains from Tugela Ferry or KwaZulu-Natal (*7**,**10*). The patient’s isolate differed by only a single locus from the F15/LAM4/KZN 605 reference strain, whereas the 4 other strains of this genotypic family varied by 1 to 3 loci (Table). Single-locus variation is prognostic of a close relationship within a clonal complex with confidence exceeding 99% (*9*). Furthermore, the patient’s isolate genotype also specifically best matched that of an F15 strain (also differing by 1 locus), in a database of 209 South African isolates containing a large variety of genotypic families (*11*).

**Table:** Locus typing for tuberculosis patient isolate according to standard nomenclature based on chromosomal locations*

Genotypic family and strain or isolate	Allele at MIRU-VNTR locus														
154	577	580	802	960	1644	2059	2165	2461	2531	2687	2996	3007	3192	4348
F15/LAM4/KZN															
Patient isolate	1	4	3	4	4	3	2	2	2	6	1	5	3	3	2
605 (reference)	1	4	2	4	4	3	2	2	2	6	1	5	3	3	2
1435	1	4	2	5	4	3	2	2	2	6	1	5	3	3	2
R506	1	4	2	3	3	3	2	2	2	6	1	4	3	3	2
V2475	1	4	2	3	3	3	2	2	2	6	1	4	3	3	2
4207	1	4	2	3+4	3+4	3	2	2	2	6	1	4+5	3	3	2
F11/LAM															
F11	2	4	2	3	3	3	2	2	2	6	1	4	3	3	2

*Patient’s isolate most closely matches F15/LAM4/KZN605 reference strain by a single locus variation, a genetic distance equal to or lower than that separating other known F15/LAM4/KZN strains (i.e., 1–3 locus variations). MIRU-VNTR, mycobacterial interspersed repetitive units–variable number tandem repeats.

## Conclusions

The risk for TB among health care workers in developing countries is well recognized (*12*) but has become more of a public health concern with evidence of the nosocomial transmission of MDR and XDR TB in South Africa. Nosocomial transmission of drug-resistant TB has been reported from well-resourced settings (*13*), but this case highlights how migration of health care workers can link the 2 settings.

When taken together, the clinical and molecular epidemiologic data in this case support the hypothesis that infection was acquired in South Africa and most likely while the patient was based in Tugela Ferry. Health care workers in South Africa are significantly more likely to be admitted to a hospital with MDR or XDR TB than are population controls (*2*), and this patient had prolonged occupational exposure at a hospital strongly associated with drug-resistant TB. Although F15/LAM4/KZN strains are frequent in South Africa, MDR versions of this genotype emerged in the KwaZulu-Natal Province in the mid-1990s, at the same time that the prevalence of HIV was increasing to hyperendemic levels and before antiretroviral therapy was widely available (*8*). The fact that this patient’s isolate and the XDR strains from Tugela Ferry have different *rpoB* mutations suggests that these strains arose independently from the locally circulating F15/LAM4/KZN strain pool, as already suggested for other MDR F15/LAM4/KZN strains (*7*).

Since this patient left South Africa, multidrug resistance has continued to grow as a challenge to public health, fueled in part by the HIV epidemic (*14*) and despite greater availability of HIV treatment. Given the long latent period between infection and the time when patients seek care for symptoms, it is reasonable to expect that such cases may become increasingly common outside Africa. If they do become more common, this change has implications for diagnostics, clinical management, and public health policy. In settings where prevalence is low but availability of resources is high, such as Europe and the United States, access to molecular diagnostic testing remains variable despite recent advances (*15*). The INNO-LiPA Rif.TB assay used here provided useful information with an indication of potential multidrug resistance. However, despite early molecular testing, identification of MDR or XDR TB still depended on conventional methods of susceptibility testing. Potentially toxic medications could have been avoided if more extensive molecular testing for resistance to second-line drugs (particularly quinolones and aminoglycosides) were achievable in close physical proximity to the patient.

The poor outcome for MDR and XDR TB in HIV-positive patients is well recognized in disease-endemic settings (*3*), and this case confirms the high risk for death even in well-resourced settings. This case also highlights deficiencies in screening policies at national borders and within occupational health, particularly for health care workers not in permanent employment. Barriers to HIV and TB testing are multifactorial, and TB screening methods have limitations; nonetheless, in this situation, there were several missed opportunities for diagnosis of TB and HIV that could have prevented this patient’s death.

This report serves as a reminder that XDR or MDR TB and HIV in sub-Saharan Africa represent not just a regional epidemic but also a challenge to international health. Although this challenge has yet to fully emerge, this case highlights the potential risk to health care workers in areas of low and high transmission.
